# Learning curves, potential and speed in training of laparoscopic skills: a randomised comparative study in a box trainer

**DOI:** 10.1007/s00464-020-07768-1

**Published:** 2020-07-08

**Authors:** Wolfgang Kunert, Pirmin Storz, Nicolaus Dietz, Steffen Axt, Claudius Falch, Andreas Kirschniak, Peter Wilhelm

**Affiliations:** 1 Department of General, Visceral and Transplant Surgery, Surgical Technology and Training, Tuebingen University Hospital, Waldhoernlestrasse 22, 72072 Tuebingen, Germany; 2 Clinic for General, Visceral and Pediatric Surgery, Duesseldorf University Hospital, Moorenstr. 5, 40225 Düsseldorf, Germany; 3 Evangelisches Krankenhaus Oberhausen, Virchowstr. 20, 46047 Oberhausen, Germany

**Keywords:** Learning curve, Learning potential, Learning speed, Laparoscopy, 3D, Stereoscopy, Knotting, Training, Randomised comparative study, Box trainer

## Abstract

**Background:**

The effectiveness of practical surgical training is characterised by an inherent learning curve. Decisive are individual initial starting capabilities, learning speed, ideal learning plateaus, and resulting learning potentials. The quantification of learning curves requires reproducible tasks with varied levels of difficulty. The hypothesis of this study is that the use of three-dimensional (3D) vision is more advantageous than two-dimensional vision (2D) for the *learning curve* in laparoscopic training.

**Methods:**

Forty laparoscopy novices were recruited and randomised to a 2D Group and a 3D Group. A laparoscopy box trainer with two standardised tasks was used for training of surgical tasks. Task 1 was a positioning task, while Task 2 called for laparoscopic knotting as a more complex process. Each task was repeated at least ten times. Performance time and the number of predefined errors were recorded. 2D performance after 3D training was assessed in an additional final 2D cycle undertaken by the 3D Group.

**Results:**

The calculated learning plateaus of both performance times and errors were lower for 3D. Independent of the vision mode the learning curves were smoother (exponential decay) and efficiency was learned faster than precision. The learning potentials varied widely depending on the corresponding initial values and learning plateaus. The final 2D performance time of the 3D-trained group was not significantly better than that of the 2D Group. The final 2D error numbers were similar for all groups.

**Conclusions:**

Stereoscopic vision can speed up laparoscopic training. The 3D learning curves resulted in better precision and efficiency. The 3D-trained group did not show inferior performance in the final 2D cycle. Consequently, we encourage the training of surgical competences like suturing and knotting under 3D vision, even if it is not available in clinical routine.

Laparoscopic surgery requires the surgeon to work under indirect vision and thus necessitates training to optimize hand–eye coordination. The aim of effective training is to develop a high learning speed with optimal final manual skills (learning plateau). Deriving learning curves (LC) from operations in clinical routine is difficult to identify and depict. A review [1] of 28 clinical studies of the learning curve in laparoscopic colorectal surgery shows a wide range of five to 310 cases needed to achieve proficiency and recommends a multidimensional assessment including CUSUM analysis. But neither the CUSUM nor the LC-CUSUM model are meant to determine a learning plateau. In fact, they need prescribed input values that define an acceptable quality or a proficient process in order to produce a binary output value, e.g. the decision whether a learning process has been completed or not. Analog to learning plateau levels, such boundary conditions are difficult to determine. The problem is that these unsafe input variables have a crucial impact on the results of a CUSUM analysis. Another review of 166 clinical studies of the learning curve in robotic surgery leads to the similar conclusion that “the outcomes reported in studies assessing LC in robotic surgery are extremely heterogeneous,” and that “despite many publications there is still no consensus […]” [2]. Therefore, in an aim to assess LC properties of basic laparoscopic skills, this study is based on performing laparoscopic tasks in a highly standardised box trainer.

A well-described parameter affecting task difficulty in laparoscopic workflows and thus learning speed is the type of endoscopic vision employed: two-dimensional (2D) vision or stereoscopic three-dimensional (3D) vision. The influence of 3D on laparoscopic surgery is well analysed and reviewed [1, 3–5]. Shortly after the first surgical 3D systems were introduced, comparative box trainer studies reported significantly faster performance with fewer errors. The clinical advantage was long disputed and 20 years later there is some consensus on a lower complication rate in clinical routine [6]. A randomised multicentre clinical trial with young surgeons showed that 3D laparoscopy was associated with reduced operative time without influencing safety [7]. However, other up-to-date randomised-controlled clinical trials [8, 9] do not confirm the benefits promised by box trainer studies. In 2017, Schwab et al. stated that universal improvement was observed when comparing 3D and 2D in studies that allowed for repetitions and plateauing of the learning curve, independent of experience [3].

Experienced surgeons with good spatial imagination develop a spatial awareness of the laparoscopic instruments for endoscopic orientation and movement. Especially for novices, stereoscopy offers more intuitive and more reliable vision than do monocular depth cues [10]. Therefore, 3D might have an effect on novices’ LC.

High-end 6 CCD 3D video systems for laparoscopy still have a comparatively heavy camera head. The relevance of a heavier camera head is arguable, especially as mechanical support devices have been established. Moreover, new 3D systems are equipped with a more compact 2 CCD design that still offers appropriate image quality. Ergonomic issues [11] and about 66% higher investment costs [12] delay the introduction of 3D systems into clinical routine. In Italian surgical units, an approximately 15% distribution rate for 3D systems was reported in 2017 [12]. It can be assumed that a large majority of surgical units worldwide still use 2D technology.

The question whether it makes sense to train laparoscopic novices under 3D vision if only 2D technology is available in their clinical environment is the subject of controversy. To assess the hypothesis that the use of three-dimensional (3D) vision is more advantageous than two-dimensional vision (2D) for the *learning curve* in laparoscopic training we present a randomised comparative study in a standardised box trainer setup.

## Materials and methods

### Equipment

As in previous studies [10], a dual-channel [13] rod lens 10-mm laparoscope (30°), a high-definition [14] 6 CCD stereo-endoscopic camera (prototypes developed by Richard Wolf GmbH, Knittlingen, Germany), and a wavelength multiplex stereoscopic monitor with passive glasses (Infitec GmbH, Ulm, Germany) were used. The stereoscopic monitor also provides 2D vision with equal image quality without the glasses.

### Stereo vision test

Since the ability to perform stereoscopic image fusion differs from person to person [15], a stereo vision test based on the random dot principle [10] was used to exclude non-stereoscopically seeing study subjects.

### Task course

Two standardised inanimate tasks were constructed of artificial materials and precisely fixed inside a box trainer developed by our working group [10, 14, 16]. This setup includes a variety of features to guarantee a high degree of reproducibility including fixed camera position and fixed monitor-to-user distance. As shown in [16], shadows cast on the background can give helpful touch control in a positioning task. To examine the effect of three-dimensionality as isolated as possible the monocular depth cue “shadowing” was minimised by diffuse indirect illumination.

**Fig. 1:**
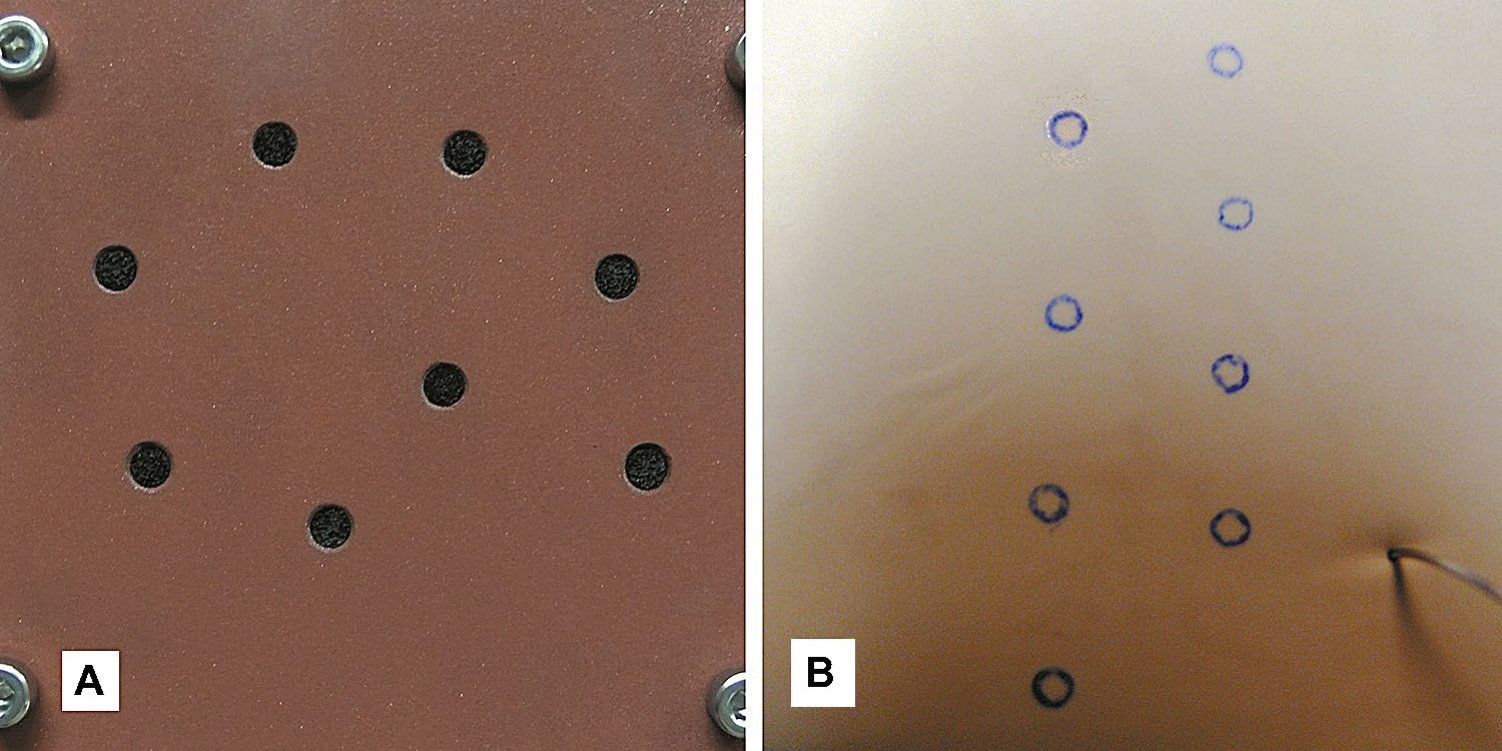
Laparoscopic views of (**A**) Task 1 and (**B**) Task 2

*Task 1* was a one-handed flat shape positioning task (Fig. 1a). Eight black circular target spots (Ø 3 mm) had to be touched once each with a Maryland dissector held in the right hand while moving in a counter-clockwise sequence. Each time the surrounding brown surface was touched, an electric circuit was closed and an error counted. Following a pilot study a number of ten task repetitions was defined to shape the LC.

*Task 2* asked for stitching and laparoscopic knot tying (Fig. 1b). A stitch had to be made through given stitch-in and stich-out points, followed by tying a knot including two reverse twists. The stitch-in and stitch-out marks (Ø 1.5 mm) were printed in ideal orientation on a disposable glove pulled over a sponge block. Because of the higher task complexity 11 repetitions were regarded as appropriate for LC assessment.

### Instruction and adaptation

The instructions for Task 1 and Task 2 were given by a recorded video that presented all necessary information in the form of a step-by-step guide. For Task 2 first a precisely defined guide for standardized laparoscopic knot tying was developed. Because of the high complexity of this task for subjects without any laparoscopic experience, a poster illustrating all necessary steps with descriptive titles was available to them at all times.

A *pick-and-place task* was integrated to smooth the switch from 3 to 2D vision. The subjects had to successively pick four pins from one box with the left-hand instrument (forceps), pass them to the right-hand Maryland dissector and place them in a different box. To avoid bias like a training effect, this adaptation task was short and simple.

### Data assessment

For both tasks the total performance times *t*_tot_ were timed digitally in microseconds, and predefined errors were countered to score precision. For Task 2 four types of errors were defined and added together to give a total error number *e*_tot_: (1) extra stitching attempt (2) stitch outside limit circle (3) incorrect knotting position (4) untight knot. To avoid a bias caused by a potential fatigue curve overlaying the LC, a pre-evaluation was performed to evaluate maximum non-stop performance time. As a result, a systematic break schedule was implemented for all subjects. The 3D Group underwent an adjunctive cycle of 2D testing at the end of each task to answer the question how persistent 3D-acquired skills are under 2D vision, which is the reality in a large number of surgical wards.

### Recruitment

The study subjects were recruited from medical students without any laparoscopic experience. The subjects volunteered without being or feeling coerced to participate. For the non-invasive study outside the hospital no formal approval by the ethics committee (institutional review board) was needed in compliance with the World Medical Association Declaration of Helsinki.

### Study design

**Fig. 2:**
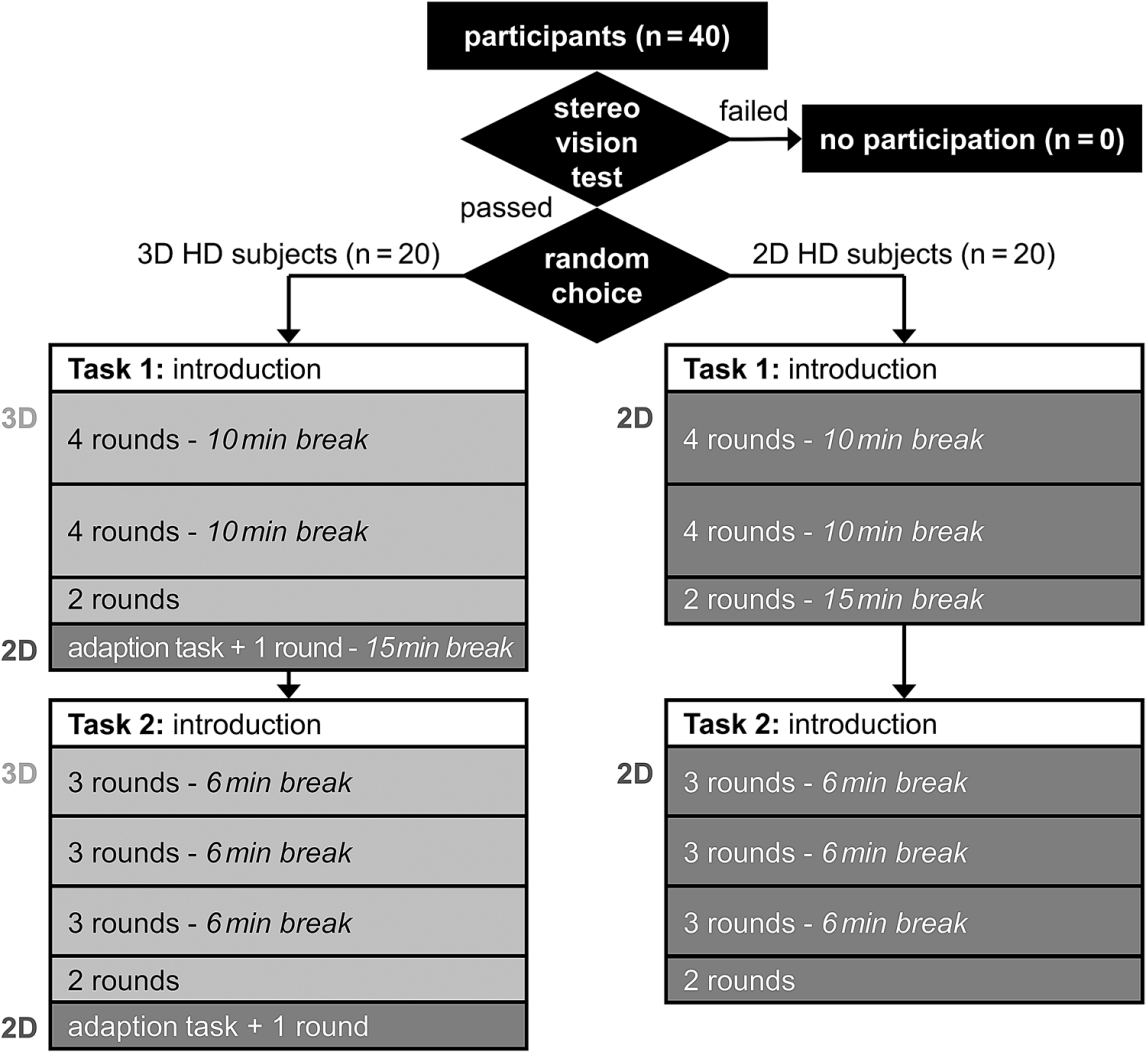
Flow chart of study design

The study compared two randomised groups, 3D vs. 2D, following the study design shown in (Fig. 2). After having passed the stereo vision test the 40 subjects were randomly divided into two groups, the 3D subjects (*n* = 20) and the 2D subjects (*n* = 20). For LC measurement, basically each subject had to perform a total of ten repetitions of Task 1 followed by 11 repetitions of the more complex Task 2. With the systemized break schedule and the additional 2D tests in the 3D Group the detailed procedure was as follows: after randomization all subjects started with the video instructions for Task 1, according to which ten training repetitions of the task had to be performed under either 2D or 3D vision with 10-min breaks after the 4th and 8th repetition. Subsequently, the 3D subjects had to perform the additional adaptation task (which was not graded) under 2D vision and repeated Task 1 under 2D vision. The same procedure was repeated for Task 2 with the difference that the intermediate breaks were only six minutes long and were taken after the 3rd, 5th and 9th repetition.

### Study endpoints

The primary study endpoint was to quantify the impact of 3D vs. 2D on the time LCs. Similarly, the secondary endpoint was to compare the error LCs.

### Forgetting and learning curves

**Fig. 3:**
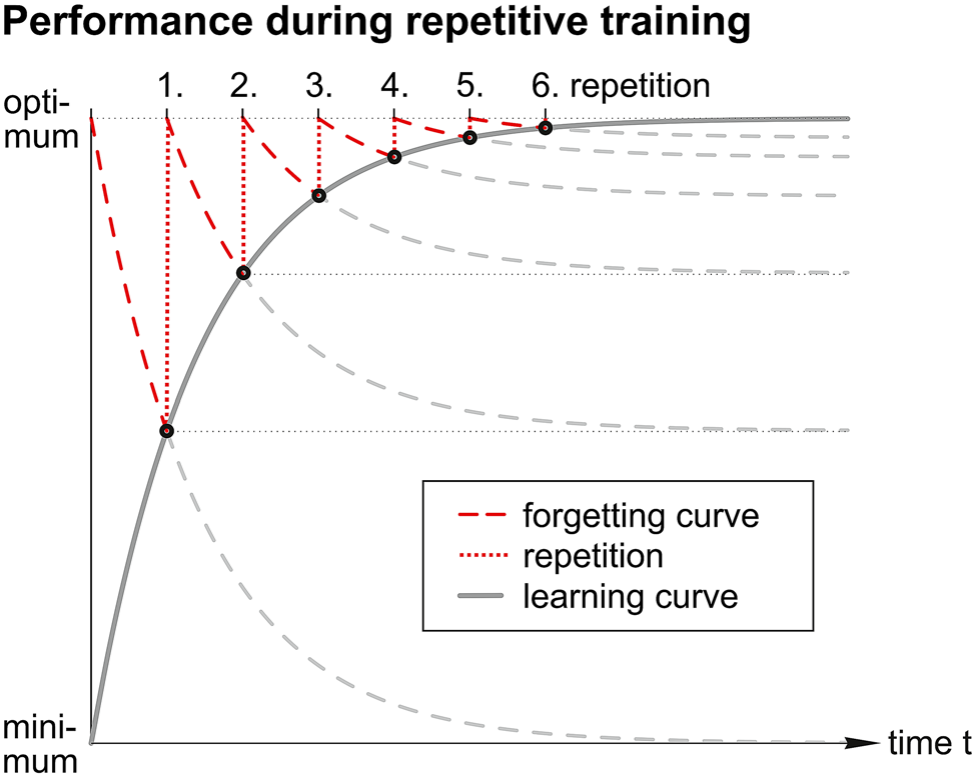
Hypothetical forgetting curves, repetitions and resulting learning curve

Repetition as a means of preventing forgetting was described 1885 by Ebbinghaus [17] in the context of learning nonsense syllables. He found that forgetting curves converge asymptotically to a minimum value and that, following repetition, LCs converge asymptotically to an ideal learning plateau value. We transferred this principle to the training of surgical skills. Figure 3 illustrates a hypothetical learning curve with a 50% forgetting rate between two subsequent repetitions. In this study the LCs are described as median total performance times and median error numbers plotted against the repetition number. For mathematical characterization an exponential decay regression curve [Formula 1] was approximated in each set of successive values. The shape of this type of idealised LC is described by the following three parameters: (1) the plateau value *y*_0_ reached after infinitely many repetitions (2) the learning potential *A*_1_ (idealised improvement) defined as the difference of the initial minus the plateau value and (3) the learning speed *t*_1_. As seen from Formula 1, *t*_1_ is a dimensionless value that compares the learning speed and the number of repetitions. When describing performance times, a high absolute value for the learning speed *t*_1_ indicates slow learning and a low absolute value for *t*_1_ indicates fast learning. For example, a typical absolute value of *t*_1_ = 3 means that 95% of the learning potential is learned with the 10th repetition.

Formula 1: Exponential decay regression formula:

$$y = y_{0} + A_{1} e^{{\frac{{1 - x}}{{t_{1} }}}}$$

*Y *= exponential decay regression, x = number of repetition, *y*_0_ = plateau value for *n* = ∞, *A*_1_ = learning potential *y*(1)−* y*(∞), *t*_1_ = learning speed.

### Statistical analysis

For statistical analysis SPSS® version 25 (IBM, Armonk, NY, USA) software was used. All groups were analysed for normal distribution using the Shapiro Wilk W test. The Mann–Whitney *U* test was applied to compare the final 2D cycle performed by the 3D subjects and the last repetition performed by the 2D subjects. *P* values < 0.05 were considered significant. For the exponential decay fit, Origin® (Microcal Inc., Northampton, MA, USA) was used.

## Results

**Fig. 4:**
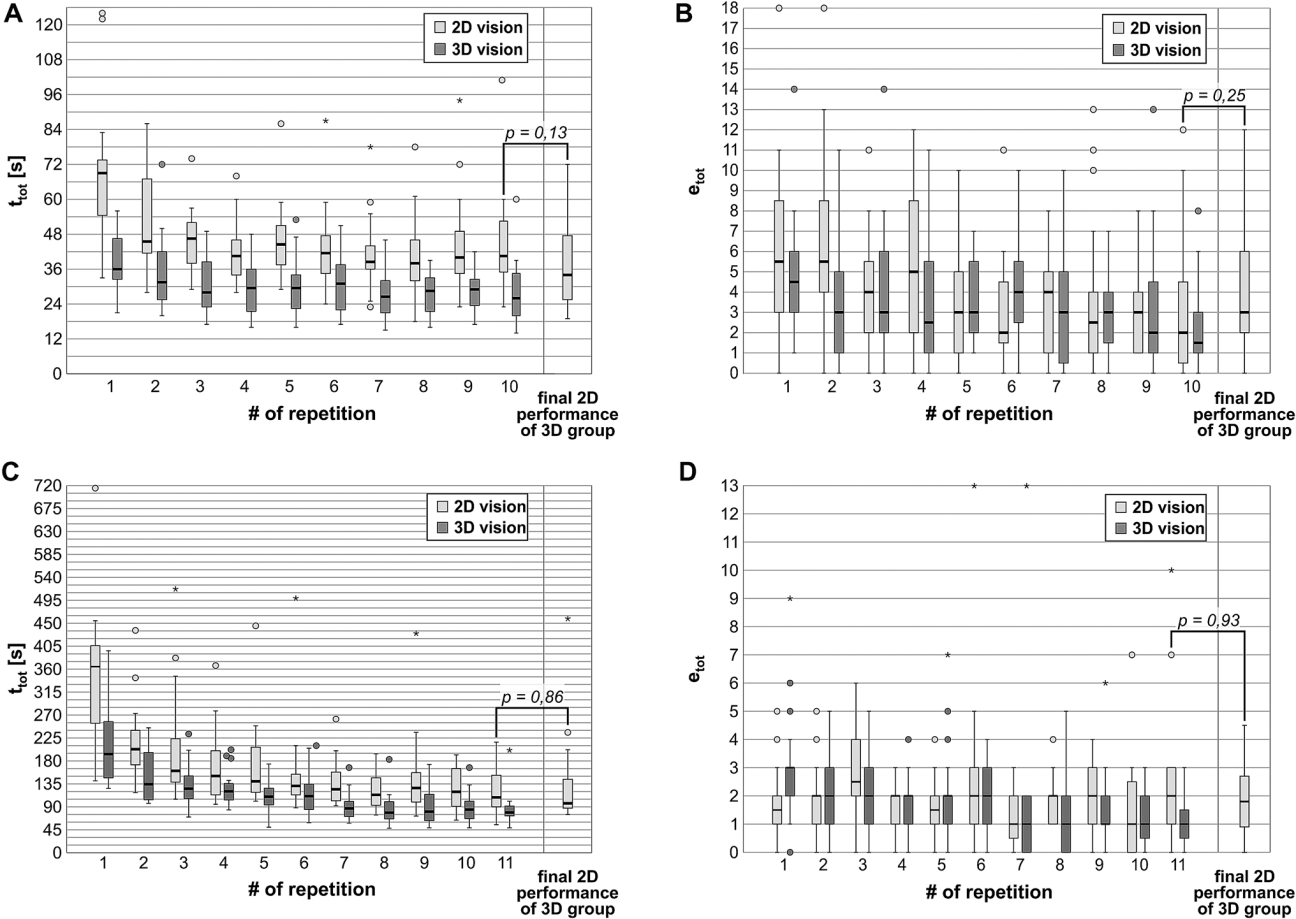
Median total performance times *t*_tot_ and total error numbers *e*_tot_. Horizontal bands indicate medians, boxes indicate Tukey percentiles, circles indicate mild outliers (1.5 IQA to 3 IQA), stars indicate extreme outliers (> 3 IQA), and whisker lines indicate highest and lowest values excluding outliers. **A** *t*_tot_ to complete Task 1 [s], **B** *e*_tot_ for Task 1, **C** *t*_tot_ to complete Task 2 [s], **D** *e*_tot_ for Task 2. *n* = 40 subjects

**Fig. 5:**
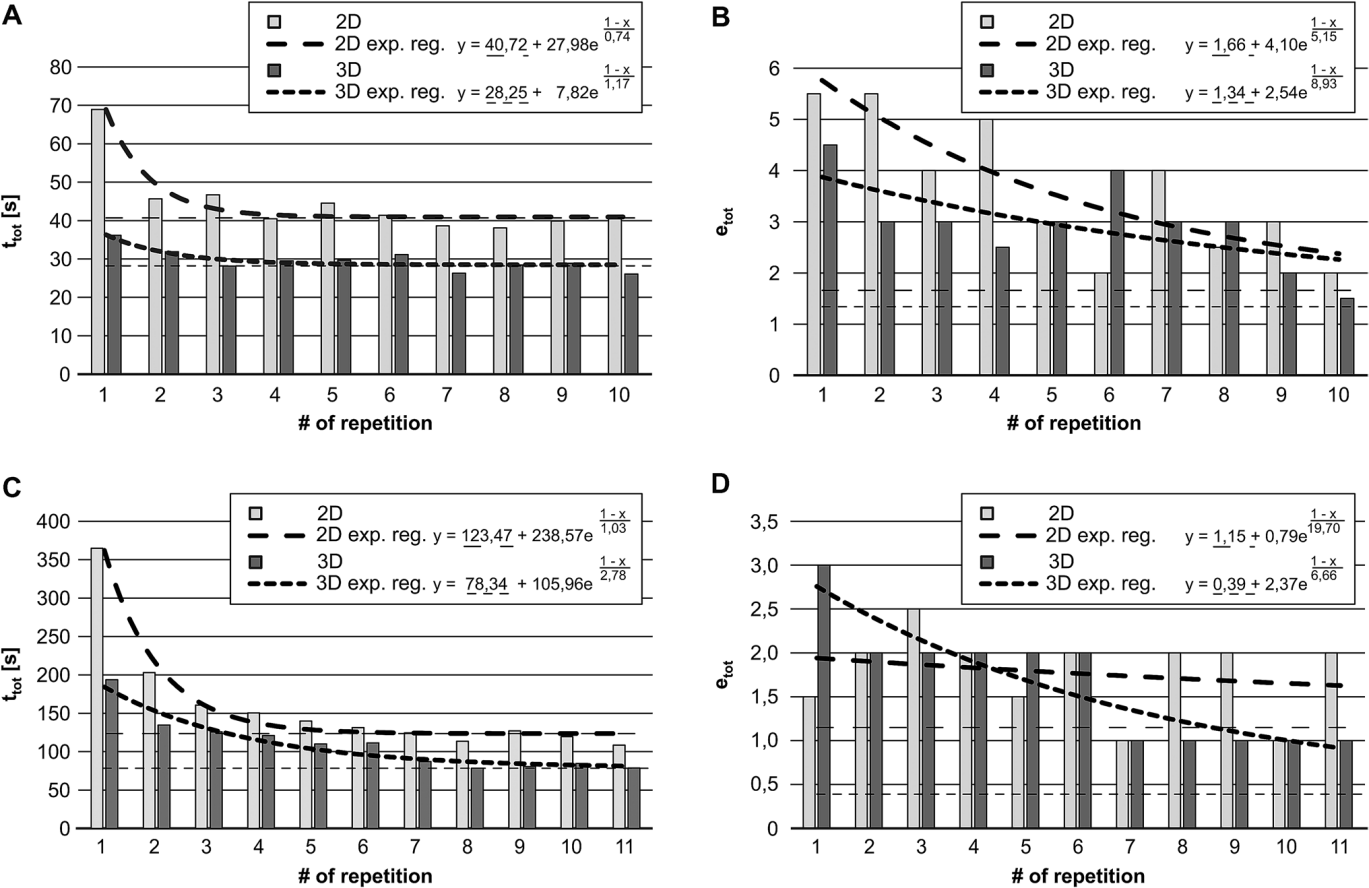
Learning curves indicated by exponential decay regression (fat lines) and plateau levels (thin horizontal lines). **A** *t*_tot_ to complete Task 1 [s], **B** *e*_tot_ for Task 1, **c** *t*_tot_ to complete Task 2 [s], **D** *e*_tot_ for Task 2. *n* = 40 subjects

The measured total performance times *t*_tot_ and total error numbers *e*_tot_ were in general distribution-free (leaning to the right). Table 1 lists the subjects’ median *t*_tot_ and *e*_tot_ for both tasks. Figure 4 shows the descriptive statistics of *t*_tot_ and *e*_tot_ in the shape of boxplots and compares the final 2D performances (Task 1: *p*(*t*_tot_) = 0.13, *p*(*e*_tot_) = 0.25; Task 2: *p*(*t*_tot_) = 0.86, *p*(*e*_tot_) = 0.93). Figure 5 depicts the median values for *t*_tot_ and *e*_tot_ with the plotted LCs and learning plateau levels.

**Table 1:** Study subjects’ median total performance times t_tot_ and median total error numbers *e*_tot_ in Task 1 and Task 2

Task 1	Median performance times [s] of		Median # of total errors of	
	2D subjects	3D subjects	2D subjects	3D subjects
Repetition 1	69.0	36.1	5.5	4.5
Repetition 2	45.7	31.9	5.5	3
Repetition 3	46.7	28.1	4	3
Repetition 4	40.5	29.5	5	2.5
Repetition 5	44.5	29.7	3	3
Repetition 6	41.4	31.1	2	4
Repetition 7	38.6	26.3	4	3
Repetition 8	38.1	28.4	2.5	3
Repetition 9	39.9	28.9	3	2
Repetition 10	40.5	26.1	2	1.5
2D-Cycle		34.1		3
*Exponential decay regression*				
Plateau *y*_0_ [s]	40.72	28.25	1.66	1.34
Learning potential *A*_1_ [s]	27.98	7.82	4.10	2.54
Learning speed *t*_1_	0.74	1.17	5.15	8.93
RMSD [s]	2.21	1.45	0.70	0.62
RMSD/*A*_1_ [%]	7.8	18.6	17.0	24.6

Task 2	Median performance times [s] of		Median # of total errors of	
	2D subjects	3D subjects	2D subjects	3D subjects
Repetition 1	364.8	193.7	1.5	3
Repetition 2	202.8	134.6	2	2
Repetition 3	160.6	125.5	2.5	2
Repetition 4	150.5	120.8	2	2
Repetition 5	139.8	109.9	1.5	2
Repetition 6	131.1	111.4	2	2
Repetition 7	124.6	87.0	1	1
Repetition 8	113.5	78.5	2	1
Repetition 9	127.0	80.8	2	1
Repetition 10	119.4	84.5	1	1
Repetition 11	108.3	78.7	2	1
2D-Cycle		96.9		2
*Exponential decay regression*				
Plateau *y*_0_ [s]	123.47	78.34	1.15	0.39
Learning potential *A*_1_ [s]	238.57	105.96	0.79	2.37
Learning speed *t*_1_	1.03	2.78	19.70	6.66
RMSD [s]	8.85	8.81	0.43	0.27
RMSD [s]	3.7	8.3	55.1	11.4

Parameters of the found LCs characterized by exponential decay regression functions

2D subjects = study participants training with a 2 dimensional high resolution vision system, 3D subjects = study participants training with a 3 dimensional high resolution vision system, RMSD = root-mean-square deviation: the lower the RMSD value, the better the regression fits

### Learning curves

For characterisation of the LCs, Formula 1 (exponential decay regression) was used. The parameters and the root mean square deviations (RMSD) indicate the quality of fit (Table 1). In three cases the 3D LC is completely below the 2D LC. In Task 2 the error LCs intersect at the 4th repetition. With every repetition the 3D subjects performed significantly faster than the 2D subjects (Task 1: median time saving 13 s, *p* < 0.001; Task 2: median time saving 35 s, *p* < 0.05). There was no significant difference in error count (Task 1: median error reduction with 3D = 1; Task 2: median error reduction with 3D = 0).

### Learning plateaus

For both tasks all learning plateaus were lower (better) under 3D vision (Task 1: *t*_tot_ = 28.25 s, *e*_tot_ = 1.34; Task 2: *t*_tot_ = 78.34 s, *e*_tot_ = 0.39) than under 2D vision (Task 1: *t*_tot_ = 40.72 s, *e*_tot_ = 1.66; Task 2: *t*_tot_ = 123.47 s, *e*_tot_ = 1.15).

### Learning potential

The *t*_tot_ learning potential was higher in the 2D Group (Task 1: *A*_1_ = 27.98 s; Task 2: *A*_1_ = 238.57 s) than in the 3D Group (Task 1: *A*_1_ = 7.82 s; Task 2: *A*_1_ = 105.96 s). The *e*_tot_ learning potential was also higher in the 2D Group for Task 1 (2D: *A*_1_ = 4.10 s; 3D: *A*_1_ = 2.54 s), but in the 3D Group it was higher for Task 2 (2D: *A*_1_ = 0.79 s; 3D: *A*_1_ = 2.37 s).

### Learning speed

The *t*_tot_ learning speed was higher in the 2D Group (Task 1: *t*_1_ = 0.74; Task 2: *t*_1_ = 1.03) than in the 3D Group (Task 1: *t*_1_ = 1.17; Task 2: *t*_1_ = 2.78). The *e*_tot_ learning speed was also higher in the 2D Group for Task 1 (2D: *t*_1_ = 5.15; 3D: *t*_1_ = 8.93), but higher in the 3D Group for Task 2 (2D: *t*_1_ = 19.70; 3D: *t*_1_ = 6.66).

### Final 2D performance

In the final 2D cycle the 3D-trained group (Task 1: *t*_tot_ = 34.1 s, Task 2: *t*_tot_ = 96.9 s) was faster for both tasks, although not significantly, than was the 2D Group (Task 1: *t*_tot_ = 40.5 s, Task 2: *t*_tot_ = 108.3 s). For Task 1 the 2D Group had a better final 2D total error number (*e*_tot_ = 2) than did the 3D Group (*e*_tot_ = 3). For Task 2 both groups had the same final 2D total error number (*e*_tot_ = 2).

## Discussion

### Limitations and biases

This study was limited to laparoscopic novices in an inanimate standardized box trainer setting. The transferability of study results from the laboratory to clinical routine is difficult, just as their transfer from novices to experts. This study has the risk of biases because its two arms are not equal. One bias is that adaptation problems between 3 and 2D could have resulted in a negative bias in the 3D subjects after Task 1. A second bias is that the 3D arm had one additional task, the 2D cycle after Task 1, to verify whether 3D learning is valuable for future 2D routine surgery. This additional 2D cycle could possibly result in an additional training session for Task 2 in favour of the 3D Group. The latter bias could have been reduced by adding another 2D repetition to the 2D arm. The authors abandoned the option of the additional 2D repetition, bearing in mind that these two possible biases compensate each other. In addition, Task 2 was very different and more demanding than Task 1. Therefore, we decided to accept the possible cross-over effect.

### Parameters of the extracted learning curves (LC)

The LCs of this study showed a good fit for the *exponential decay regressions* with respect to times *t*_tot,_ but only partly good with respect to errors *e*_tot_. The round-off errors deriving from the used median values that are ordinally and not interval-scaled make a contribution to the limited fit quality of the error LCs. A better fit results when based on an adapted staircase-like LC. However, with regard to errors it is doubtful that any learning occurred in the 2D Group for the more complex Task 2. In all other cases the initial time and error values were lower in the 3D Group. Consequently, in these other cases the 3D LCs were completely below the corresponding 2D curves, indicating a general advantage of 3D over 2D. Performance by the 3D high-definition (HD) subjects was faster at all points of their LC than was performance by the 2D HD subjects. Therefore, 3D subjects reached the same performance level in an earlier repetition.

*Learning potentials* varied widely depending on the corresponding initial values and learning plateaus. *Learning speed* in the 2D Group was generally higher, but was not an advantage, because it could only partly catch up with the generally much better starting conditions of the 3D Group. Contrarily, in all cases the final results and the calculated *learning plateaus* were better in the 3D Group than in the 2D Group. In most cases some learning benefit of the 3D training continued, even in the final 2D cycle. The two learning plateaus of the more complex intracorporeal suturing and knotting Task 2 were better than were those of the positioning Task 1. This finding supports the EAES consensus, which states that 3D is more advantageous for clinically complex tasks [6]. Interestingly, knot quality improved much more slowly than time efficiency. This suggests that teaching should be improved and encourages the conservative surgeon to put quality first, even if it takes longer.

### Comparison with other studies

Verdaasdonk [19] had six virtual pick-and-place tasks repeated ten times using the SIMENDO simulator. The participants were divided into four groups having different levels of experience with endoscopic surgery from novice to expert. The four quality parameters were task time, number of collisions, endoscope path length and right instrument path length. The resulting medians of the data were plotted over the repetition number, thus creating 16 LCs. An illustration of a theoretical concept of LCs is given, but the measured LCs were not further analysed. Although, in contrast to our study, these LCs “only” emerged from virtual reality, they show striking similarities to the curves observed in our study in the box trainer. The LCs for the task time look especially similar to the LC (*t*_tot_) for Task 2 in our study and probably could be approximated well in an exponential decay regression. The number of collision LCs looks rather like that of our error LCs (*e*_tot_). Therefore, both simulator and box trainer give reproducible measures. “However, these reproducible measures alone are insufficient to demonstrate transferability of skills from the laboratory to the operating room,” concluded Buckley et al. [18] after reviewing 16 controlled trials with OSATS scores using the surgical simulation systems LapSim, MIST-VR, LapMentor, MISTELS, SCMIS GEM or SIMENDO.

De Win [20] reports a randomized simulation study including 30 novices. The subjects were divided into three types of training groups and ten parameters were measured or derived to create a total of 30 LCs. Because of the small number of five repetitions it is difficult to interpret the character of the LCs. Still, the time-related LCs are slightly convex and include an asymptotic course. As in the error LCs of our Task 2, the LC plots of some parameters show intersections. In these six cases the error indicators suggest that the learning effect had only a small impact on the plotted parameter as compared to the accuracy of the measurement.

Kyriazis [21] examined the operative time LC after switching from 2 to 3D vision in five consecutive patients undergoing laparoscopic extraperitoneal radical prostatectomy by an experienced surgeon. Although the expert learned in the clinic, a slightly convex potentially asymptotic LC still resulted. Unfortunately, there was no switching from 3 to 2D vision to examine the question of 3D skills in a 2D surgical unit.

Laubert [22] performed the Lübeck Toolbox Curriculum by recruiting 63 novices who repeated six different standardised tasks up to 80 times under 2D vision. Plateau values were estimated based on the performance of experts. The not further analysed six LCs seem to follow very well an exponential decay function.

Rosser [23] measured performance times when repeating the four tasks “rope pass”, “cup drop”, “triangle transfer” and “intracorporeal suturing” at least ten times solely under 2D vision. A significant (*p* < 0.001) improvement is reported for a comparison of the initial and the last repetition of all tasks, but the shapes of the LCs are not characterised. However, they also seem to follow an exponential decay function. Rosser’s “intracorporeal suturing” performance is highly similar to Fig. 5c, starting with an initial value of 376.3 ± 18.33 s, ending after the 11th repetition with a final value of 150.75 ± 2.95 s and showing almost continuous improvement.

Kong [24] report a comparative study of 2D and 3D vision with four repetitions for the two tasks “threading through rings” and “dividing and cut”. Two groups, novices and experienced surgeons, were engaged for four consecutive days. With the aim of balancing out the learning effect, the subjects alternately worked under 2D and 3D vision. Nevertheless, Kong reported a time and an error LC, both with a greater learning effect for the novices and a lesser learning effect for the experienced group. Because the 2D and the 3D Groups in our study were not independent their results can not be compared with those of our study. With only four repetitions it is hardly possible to extract a LC.

Blavier [25] compared four groups of novices (2D robot, 3D robot, 2D laparoscopic, direct view 3D laparoscopic) performing a “threading through rings” task. The task was repeated six times under one vision mode. Then the vision mode was switched and the task was repeated another two times. Different scores were recorded for errors, ambidexterity and performance. Contrary to our study, Blavier found significant deteriorations after the vision switch, regardless of whether the switch went from 3 to 2D or vice versa. One possible reason is that the task was much more focused for absolute 3D positioning than was our knotting task. Another possibility is that enough learning did not take place during the six repetitions and the LC was still too steep to ensure a permanent learning effect. In our task scenario it looks like the subjects undergoing 3D training developed a compensating mechanism for orientation in space. Because of the minimized monocular depth cues in our setup (e.g. almost no shadowing), it seems that this compensation mechanism is primarily based on proprioceptive spatial perception. In combination with motion memory learning of movements this could be especially helpful for mastering repetitive tasks like endocorporeal knotting.

## Conclusion

Analysis of this study revealed learning curves (LCs) in the shape of exponential decay curves. Also many other training data sets found in the literature seem to describe well this type of curve. To obtain a good fit interval-scaled data are preferable. According to this study, stereoscopic 3D vision can speed up laparoscopic training. The 3D LCs can result in better precision and efficiency as compared to 2D, or the same result can be achieved after fewer repetitions. The 3D-trained group did not show inferior performance in the final 2D cycle. Consequently, we encourage the training of mechanical workflows like suturing and knotting under 3D vision, even when it is not available in clinical routine.
